# Effects of glaucoma drugs on ocular hemodynamics in normal tension glaucoma: a randomized trial comparing bimatoprost and latanoprost with dorzolamide [ISRCTN18873428]

**DOI:** 10.1186/1471-2415-5-6

**Published:** 2005-04-05

**Authors:** Oliver Zeitz, Eike T Matthiessen, Juliane Reuss, Anne Wiermann, Lars Wagenfeld, Peter Galambos, Gisbert Richard, Maren Klemm

**Affiliations:** 1 Universitätsklinikum Hamburg-Eppendorf, Klinik und Poliklinik für Augenheilkunde, Martinistr. 52, D- 20246 Hamburg, Germany

## Abstract

**Background:**

Reduced choroidal perfusion is hypothesized to play a role in the pathogenesis of normal tension glaucoma. Thus the impact of antiglaucomatous eye drops on ocular perfusion has been the focus of recent research and the subject of intensive investigations. The present study investigates whether topically applied latanoprost or bimatoprost influence ocular perfusion in patients with normal tension glaucoma and compares these effects with that changes detected after the treatment with dorzolamide.

**Methods:**

Ocular hemodynamics were assessed by color Doppler imaging (CDI) shortly before and after a one-month treatment with either latanoprost, bimatoprost or dorzolamide. Primary end-points of the study were peak systolic and end-diastolic blood flow velocities in the short posterior ciliary artery (SPCA) under the new therapy. Intraocular pressure (IOP) and additional perfusion parameters in the SPCA and other retrobulbar vessels were tracked as observational parameters. n = 42 patients with normal tension glaucoma were enrolled in the study.

**Results:**

Systolic and diastolic blood flow velocities in the SPCA showed no significant alteration after the treatment with latanoprost or bimatoprost. Dorzolamide lead to increase of peak systolic velocity. IOP was reduced by all three agents in a range reported in the literature.

**Conclusion:**

Topically applied latanoprost and bimatoprost act in a hemodynamically neutral manner and have the capability to lower IOP even in patients with normal tension glaucoma and low initial IOP level. Dorzolamide accelerates blood flow in systole. None of the tested compounds has a negative impact on hemodynamics in the short posterior ciliary arteries.

## Background

Traditionally atrophy of the optic nerve head in glaucoma patients has been related to a chronically increased intraocular pressure (IOP).[1] In the majority of glaucoma patients increased IOP levels can be measured. At the same time there is a significant number of patients who develop the major features of glaucoma, meaning progressive visual field loss and atrophy of the optic nerve head, although the IOP is in the normal range below 21 mmHg in these individuals. [2-6] This variant of primary open angle glaucoma has been termed as normal tension glaucoma and the glaucomatous changes have been – at least in part – attributed to reduced perfusion of the optic nerve head by different authors. [6-8] Thus patients with normal tension glaucoma constitute a particularly interesting group for investigating pharmacological effects on ocular perfusion.

Up to now therapy of normal tension glaucoma does not differ substantially from the therapy of classical primary open angle glaucoma with increased IOP level: in both cases the focus is set on lowering IOP.[9,10] There is a tendency for defining lower target IOPs individually for each patient and optimizing ocular hemodynamics is still rated as complementary. Recently, several systemic approaches have been evaluated, e.g. oral administration of magnesium or the calcium antagonist nimodipine.[11,12] In addition, the hemodynamic effects of locally applied antiglaucomatous drugs are subject to intensive investigations within the last years: [13-16] Dorzolamide, an inhibitor of the carbonic anhydrase has shown the most promising effects on ocular hemodynamics. [17-19] In contrast, brinzolamide did not to alter ocular perfusion.[20] ß-receptor antagonists influence parameters of ocular hemodynamics differently depending on the compound used: betaxolol and carteolol lower vascular resistance while timolol seems to lead to an increased vascular tone.[15] The α_2_-receptor agonist brimonidine did not affect ocular perfusion in primary open angle glaucoma patients.[21] Derivatives of prostaglandins have become more and more popular for the treatment of glaucoma patients since they are very effective in lowering IOP and have to be applied only once a day.[22] Up to now there are predominantly four substances in clinical use: latanoprost, bimatoprost, travoprost and unoprostone. Only few studies have addressed the influence of prostaglandin analogues on ocular perfusion so far.

Prostaglandins constitute a large group of compounds produced endogenously with various and partially contrary effects on vascular tone.[23,24] Certain prostaglandins, such as PGI_2 _and PGE_2_, are potent vasodilators; others like PGF_2α _constricts arteries. [25-27] Investigations of the vasoactive effects of prostaglandins are complicated by the species dependency of their effects on vascular tone.[28] Latanoprost derives from PGF_2α _and has been reported to improve the perfusion of the optic nerve head[13], although *in vitro *studies indicate that latanoprost in contrast to its chemical origin PGF_2α _exerts no or minimal effects on vascular tone in the primate eye.[28] Bimatoprost has been demonstrated to have a vasoconstricting effect on isolated porcine ciliary arteries *in vitro*.[29] It is unknown whether this effect is of relevance *in vivo *and in humans.

The aim of the present study is to compare the hemodynamic effects of bimatoprost and latanoprost in patients with normal tension glaucoma. The effects were compared with the hemodynamic properties of dorzolamide, which has repeatedly been shown to improve ocular blood flow and can therefore be used as a reference compound for the evaluation of hemodynamic effects of antiglaucomatous eye drops. [17-19] It is hypothesized that these compounds lead to changes in systolic and diastolic blood flow velocities within the short posterior cilary artery. This vessel constitutes the main blood supply to the optic nerve head and alterations in its perfusion might play an important role in the pathogenesis and progression of normal tension glaucoma.

## Methods

The study was performed in accordance to institutional, national, and international guidelines and was approved by the local ethics committee. The study was designed as an interventional, randomized, prospective, institutional, single-blinded, controlled, clinical trial.

### Color Doppler imaging

Color Doppler imaging (CDI) was performed with a Sonoline Elegra Advanced System (Siemens, Erlangen, Germany) using a phased array transducer type 7.5L40 (Siemens, Erlangen, Germany) as described previously.[30] Ultrasound frequency was 6.5 MHz in the pulsed Doppler mode. The transducer was carefully set on the closed eye lid without exerting pressure on the bulb. Acoustic coupling between transducer and skin was optimized by a carbomeric gel (Vidisic^®^, Dr. Mann Pharma, Germany). After identification of the optic nerve as a landmark the following vessels were investigated in the color Doppler mode: central retinal artery (CRA), short and long posterior ciliary arteries (SPCA and LPCA) and ophthalmic artery (OA). Blood flow in the CRA was measured along its course through the optic nerve. Velocities in the SPCA and LPCA were recorded before entering the sclera. Flow velocity in the OA was measured close to its crossing of the optic nerve. The angle between transducer and orientation of the vessel was corrected. Gain and threshold were adjusted individually for each examination until noise disappeared and then kept constant during the entire examination. Pulse repetition frequency (PRF) was minimized to avoid aliasing (typically 5208 Hz for the OA, and 2500 Hz for the SPCA and LPCA in the Doppler mode). Sample size for all vessels was set constantly to 1.5 mm.

The changes of blood flow velocities over the course of a cardiac cycle were recorded continuously. Peak systolic velocity (PSV) and end diastolic velocity (EDV) can be determined directly and pulsatility index (PI) and resistive index (RI) are calculated automatically by the CDI software.

### Patients

Consecutive patients with progressive normal tension glaucoma were randomized either to the latanoprost or to the bimatoprost group. Progression was defined as progressive excavation of the optic disc (funduscopic and controlled by the Heidelberg retina tomography – HRT) and/or progressive visual field loss in the Humphrey perimeter over the last 6–12 months. Glaucoma progression was assessed by the same glaucoma specialist (M.K.) in all patients. The patients underwent CDI measurements of ocular perfusion of the right eye by CDI shortly before and 3 to 5 weeks after initiation a local therapy with either latanoprost or bimatoprost. Both eye drops were applied once a day between 6 p.m. and 8 p.m. Normal tension glaucoma patients with unchanged excavation of the optic disc in funduscopy and HRT as well as stable visual field over the last 6–12 months served as negative controls. The glaucoma parameters in the control group were assessed by the same glaucoma specialist (M.K.) like in the therapy group. In the control group two subsequent CDI examinations were performed with an interval of 3 to 5 weeks. An additional group of patients with progressive normal tension glaucoma received dorzolamide eye drops three times a day, which has been reported to enhance blood flow in retrobulbar vessels. [17-19] This group was recruited to demonstrate that the CDI-methodology used is suitable to detect changes in retrobulbar blood flow. Regarding the assessment of progression the same criteria were applied as for the other study groups. CDI-examinations in the dorzolamide group were carried out shortly before and 3 to 5 weeks after initiation of the dorzolamide therapy.

All measurements in both therapy and control groups were carried out at 4 p.m. ± 2 h as a standardized time point to ensure best possible comparability. This specific time point was chosen due to organizational reasons. Prior to CDI intraocular pressure was measured. All examinations were performed with the patient sitting in an upright position and all the patients rested for several minutes before the examination started. CDI measurements for this study were performed by two experienced investigators (O.Z. and E.T.M.). To exclude investigator dependent effects, both CDI measurements in one patient were performed by the same physician. The study group membership and the individual therapy of the patients were masked for the CDI-investigator.

Patients with an intraocular pressure higher than 21 mmHg prior to therapy as well as patients with cardiovascular diseases were not included in the study. Systemic medication of the patients did not change between their first and second presentation. Patients who had ophthalmic surgery to the right eye were excluded from the study.

### Bimatoprost

Lumigan^® ^eye drops from Pharm Allergan (Ettlingen, Germany) were used in the bimatoprost treatment group. Lumigan^® ^contains 0.3 mg/ml bimatoprost. The solvent contained benz alkonium chloride, sodium chloride, di sodium hydrogene phosphate 7H_2_O, citric acid, hydrochloric acid or sodium hydroxide (for pH adjustment), and water.

### Latanoprost

Latanoprost (50 μg/ml) was obtained from Pharmacia Pfizer (Karlsruhe, Germany) as Xalatan^®^. Further ingredients are benz alkonium chloride, sodium chloride, sodium dihydrogene phosphate 1H_2_O, sodium monohydrogene phosphate, and water.

### Dorzolamide

Dorzolamide was obtained in form of Trusopt^® ^from MSD-Chibret, Munich, Germany. Trusopt^® ^containes 22.26 mg dorzolamide hydrochloride per 1 ml. Further ingredients of Trusopt^® ^are hydroxyethylcellulose, D-mannitole, sodium citrate, sodium hydroxide, benzalkonium chloride and water as solvent.

### Statistics

Statistical analysis of the data was done with SPSS 10.0. All data are given as mean ± standard error of means (SEM). Student's t-test for paired data was used. It was hypothesized that the tested compounds would influence peak systolic and end-diastolic blood flow velocities (PSV and EDV) in the short posterior ciliary artery (SPCA). Thus two statistical tests were performed in each of the four study groups resulting in eight parallel statistical tests in total. Performing multiple statistical tests in a study necessitates the correction of the P-value to reach a significance level of 0.05. Therefore the Bonferroni-adjustment was applied and P < 0.006 is considered to be significant (double sided). All other parameters except EDV and PSV in SPCA were evaluated in a descriptive manner. Thus no P-values will be presented for these observational parameters.

In an a-priori-power-analysis the sample size was calculated. H1 was defined by an increase of flow velocity by 50% and a standard deviation of 35%. This definition was applied because preliminary data indicated a change in that range caused by dorzolamide. To reach a statistic power of 0.80 or more at least n = 9 individuals for PSV and n = 8 individuals for EDV per treatment group are required. For power analysis, the tool G*POWER V. 2.0 of F. Faul and E. Erdfelder [31] was applied.

## Results

11 patients were treated with bimatoprost, 10 patients with latanoprost. Initially 9 patients were enrolled in the negative control group, but only 8 could be evaluated. One patient had to be excluded, because he did not return to the second examination due to personal reasons. 12 additional patients received dorzolamide. All data shown was obtained from measurements of the right eye of the patients.

### Intraocular pressure (IOP)

**Figure 1:**
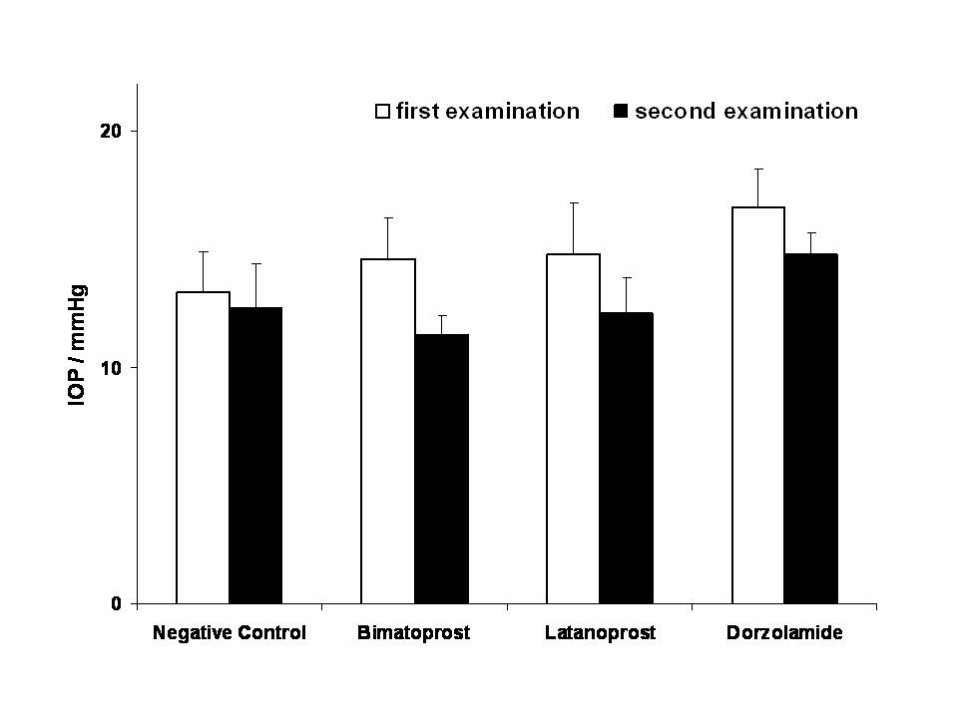
**Intraocular pressure changes**. Intraocular pressure (IOP) at first and second examination. IOP was an observational parameter and was not subjected to statistical testing.

Intraocular pressure was reduced from 14.6 ± 1.7 to 11.4 ± 0.8 mmHg under therapy with bimatoprost; latanoprost led to a decrease from 14.8 ± 2.2 to 12.3 ± 1.5 mmHg. Dorzolamide decreased the IOP in the dorzolamide group from 16.8 ± 1.6 to 14.8 ± 0.9 mmHg. In the negative control group intraocular pressure was 13.2 ± 1.7 mmHg at first presentation and 12.5 ± 1.9 mmHg at second presentation 3 to 5 weeks later (fig. 1).

### Color Doppler imaging (CDI)

**Figure 2:**
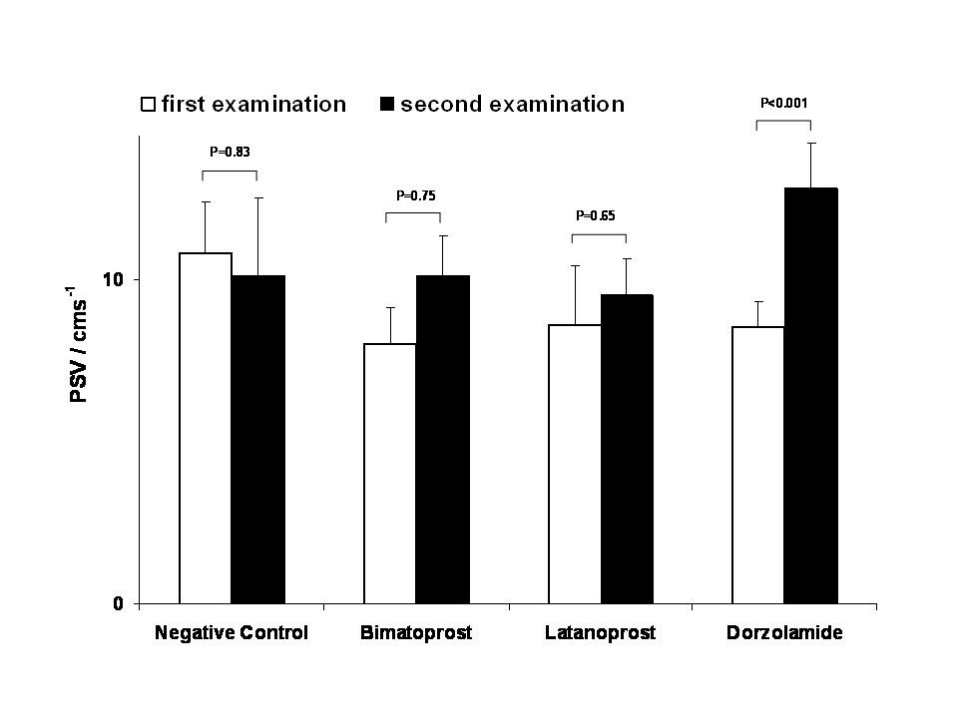
**Peak systolic velocity**. Peak systolic velocity (PSV) at first and second examination. In tendency, both compounds led to a slight increase of PSV, which was statistically not significant. Dorzolamide led to a statistical increase of PSV.

**Figure 3:**
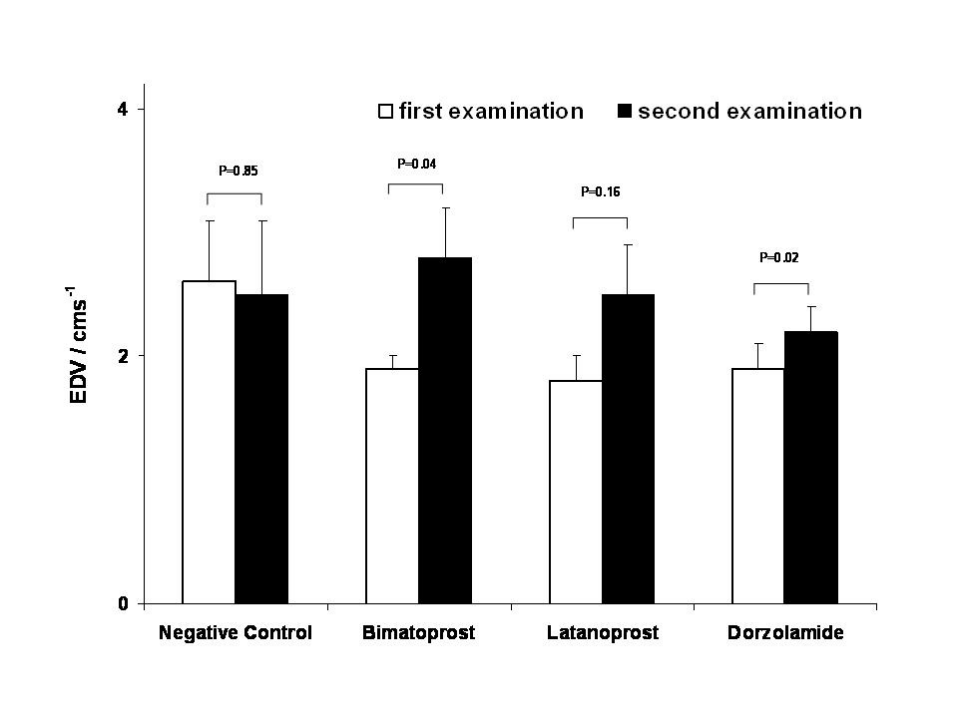
**End diastolic velocity**. End-diastolic velocity (EDV) at first and second examination. In tendency, both compounds led to a slight increase of EDV, which was statistically not significant.

Neither bimatoprost nor latanoprost showed a statistically significant influence on peak systolic or enddiastolic velocities in the short posterior ciliary artery (fig. 2 and 3). All other parameters assessed by CDI measurements in the central retinal artery, short and long posterior ciliary arteries and ophthalmic artery appeared to be stable. Table 1 gives a detailed overview of the quantitative results obtained by CDI before and after treatment with latanoprost and table 2 does this for bimatoprost. In the negative control group all parameters were stable over time (table 3). Dorzolamide lead to a significant acceleration of systolic blood flow in the short posterior ciliary artery (table 4).

## Discussion

Based on the presented results bimatoprost or latanoprost and have no significant influence on hemodynamics in the short posterior ciliary artery has to be rejected, although a tendency toward improved peak systolic and end diastolic blood flow velocities was detected in both treatment groups compared with the negative control group.

To our knowledge the present study is the first to directly compare hemodynamic effects of bimatoprost and latanoprost in patients with normal tension glaucoma. An altered blood flow velocity due to a vasoconstricting activity of bimatoprost reported from Allemann and colleagues[29] is not detectable in humans by CDI measurements. These differences might be explained by the *in vitro *system used by Allemann, who worked with isolated ciliary arteries from pigs. Apart from potential differences between species, the balance between neuronal, endothelial and myogenic influences on vascular tone is disturbed in such an *in vitro *setting. *In vitro *studies for latanoprost revealed minimal vasoactivity.[28] The chemical origin of latanoprost is the prostaglandin PGF_2α_, which increases arterial tone. [25-27] Bimatoprost is a derivative of the prostamide F_2α_, which is enzymatically formed from PGF_2α _and should act similar like PGF_2α_, but has a lower receptor affinity.[32] How vasoconstriction influences ocular perfusion depends on its site of action: Constriction of pre-capillary sphincters will cause a deterioration of blood flow. On the other hand, moderate constriction of the entire arterial system of an organ will increase perfusion pressure. From this theoretic view prostaglandins could influence ocular blood flow in positive and negative direction or both effects neutralize each other.

For latanoprost a handful of studies dealing with its effects on ocular perfusion have been published. Some of these studies state positive effects.[13,33,34] All these studies have in common that acute and short-term effects from less than an hour up to one week after application of the drug were observed. In accordance with the present results, studies with an extended follow up did not find any mid- or long-term effect of latanoprost on ocular hemodynamics.[13,35,36] A possible explanation for the difference between long- and short-term effects might be the acute reduction of intraocular pressure, which will result in an increased ocular perfusion pressure and leads to an improvement of ocular hemodynamics. For comparison with the presented results one has to point out that the previous reports were carried out with patients suffering from primary open angle glaucoma with initially increased IOP. Thus it is difficult to differentiate whether the observed improvement of ocular perfusion can be attributed to a primary effect of the active agent on the ocular vasculature or is secondary to IOP-reduction. In the setting of the present study the absolute IOP reduction in relation to ocular perfusion pressure is low and does affect ocular perfusion pressure only by approximately 5%. Retrobulbar blood flow velocities are directly proportional to ocular perfusion pressure. Subsequently, the effect of such a change in intraocular pressure on retrobulbar blood flow velocities is low, particularly compared to the changes seen after the administration of dorzolamide. Nevertheless, the tendency of an increase of retrobulbar blood flow velocity is visible in all treated groups.

Patients with normal tension glaucoma are therefore a particularly interesting group for the clinical investigation of hemodynamic effects of antiglaucomatous eye drops since results are less skewed by changes in IOP. In addition, a reduction in ocular perfusion might play a major role in the pathogenesis of normal tension glaucoma.[37] Reversal of this condition could be of great clinical benefit for this patient group and deterioration of ocular perfusion is expected to accelerate progression of glaucomatous optic neuropathy. Therefore normal tension glaucoma requires a therapy which ideally improves hemodynamics of the eye, but at least does not alter it in a negative way.

Although not primary end-point of the present study, the results indicate that latanoprost and bimatoprost have the capability to reduce IOP in patients with normal tension glaucoma, despite the fact that initial IOP levels before the treatment are low. Relative IOP-reduction in the present study is smaller with previous reports on treatment of patients with primary open angle glaucoma.[22,38,39] This is most likely due to the low initial intraocular pressure in the patient population with normal tension glaucoma. Studies on the IOP-lowering effect of prostaglandins, predominantly latanoprost, in normal tension glaucoma find an IOP-reduction in a similar magnitude. [40-42] This can be taken as an indicator for good compliance of the patients and it can be concluded that both tested compounds have the capability to reliably decrease IOP even in the lower range.

Some ophthalmologists doubt the reliability of CDI, but the reliability of the CDI method has repeatedly been shown *in vitro*[4] and *in vivo*[43]. Experiments performed by our group are in accordance with these findings.[44] The positive control with dorzolamide in the present study underlines the applicability of CDI for assessing pharmacological effects on ocular perfusion. To resolve the potential problem of investigator dependency both CDI-examinations for each patient were performed by the same investigator. In addition, the CDI procedure was performed in a highly standardized manner in our laboratory minimizing investigator dependency of the results.

The precise and clinically relevant evaluation of ocular perfusion still poses a great challenge. Quantitative determination of total ocular blood flow with a single method is not possible, but combination of different methods is proposed to give a better approximation.[45] This fact limits the explanatory power of this and other studies addressing ocular perfusion in ophthalmological diseases. Aim of the present study was to focus on glaucoma patients and it is hypothesized that glaucoma is associated with a localized disturbance of ocular hemodynamics at the optic disc. The optic disc is supplied with blood by the posterior ciliary arteries. The most reliable method for evaluation of hemodynamics in the short posterior ciliary artery is color Doppler imaging. Blood flow velocity in the short posterior ciliary arteries is influenced on the one hand by the vascular tone of the vessel itself but also by the resistance of the dependent downstream vasculature. Thus blood flow velocities in the short posterior ciliary artery reflect also the hemodynamics at the level of the optic disc. The influence of the tested compounds on perfusion of the entire eye cannot be answered by the present study.

Recently it has been shown that the correlation between measurements of ocular hemodynamics with different methodologies is limited.[30] Thus differences in outcome between studies investigating the effects of the same compound on ocular perfusion might at least partially be explained by the variability between the methodologies. Color Doppler imaging (CDI) measures blood flow velocities. Measurement of ocular blood flow by the method of Langham (LOBF) is based on assessing pulse synchronal oscillations of the IOP and calculate ocular perfusion from its amplitude by mathematical algorithms.[46] From the parameter determined by CDI only the pulsatility index correlates with the perfusion index determined by LOBF.[30] Therefore the comparison of the results of the present study e.g. with the work by Georgopoulus et al.[33] is limited.

## Conclusion

In summary no significant influence of latanoprost and bimatoprost on ocular hemodynamics was detected in the present study. In accordance with previous reports dorzolamide led to an increase of blood flow velocity in systole. A suitable compound for the treatment of normal tension glaucoma should at least act hemodynamically neutral. This minimum requirement is fulfilled by all three tested compounds. Only dorzolamide was capable to improve perfusion in the posterior ciliary arteries. When comparing both prostaglandin-like eye drops, they showed similar effects on ocular hemodynamics and IOP. Subsequently latanoprost and bimatoprost can be considered as equivalent in the treatment of normal tension glaucoma. However, in order to comprehensively treat the pathogenic disturbances leading or at least contributing to normal tension glaucoma there is still a great demand for a pharmacological compound which significantly enhances perfusion of the optic nerve head.

## Competing interests

M.K. received a grant from Pharm-Allergan (manufacturer of bimatoprost) in 2003. This grant is not related to the present study. Neither the present study nor the present manuscript is financed by this grant. There are no further commercial relationships for any author.

## Authors' contributions

OZ designed and co-ordinated the study. ETM and OZ performed the CDI measurements. JR and AW documented the data and did under supervision of OZ the statistical analysis. LW and PG were additionally involved in editing the manuscript and interpreting the data. PG contributed knowledge about basic science aspects of prostaglandin action on vasculature. GR and MK supported the other authors in interpreting the results. MK as glaucoma specialist was additionally responsible for patient recruitment and randomization.

## Pre-publication history

The pre-publication history for this paper can be accessed here:



## Figures and Tables

**Table 1:** Influence of bimatoprost on retrobulbar hemodynamics. **Influence of bimatoprost on ocular perfusion**. Overview of the results from CDI measurements before and one month after regular application of bimatoprost: Increase of blood flow velocities were statistically not significant (n = 11). **Bold **printed measures were subjected to statistical testing; all other parameters were observational only.

		PSV (cms^-1^)	EDV (cms^-1^)	RI	PI
OA	before bimatoprost	31.6 ± 3.5	3.5 ± 0.9	0.89 ± 0.02	2.76 ± 0.20
	after bimatoprost	38.5 ± 4.8	4.2 ± 0.7	0.87 ± 0.03	2.53 ± 0.21
					
CRA	before bimatoprost	7.9 ± 0.9	1.4 ± 0.2	0.79 ± 0.04	2.22 ± 0.31
	after bimatoprost	8.1 ± 0.5	1.7 ± 0.1	0.79 ± 0.02	1.90 ± 0.10
					
SPCA	before bimatoprost	**8.0 ± 1.1**	**1.9 ± 0.1**	0.71 ± 0.03	1.61 ± 0.15
	after bimatoprost	**10.1 ± 1.2**	**2.8 ± 0.4**	0.75 ± 0.01	1.66 ± 0.05
					
LPCA	before bimatoprost	14.8 ± 3.0	3.0 ± 0.7	0.78 ± 0.03	1.90 ± 0.17
	after bimatoprost	16.8 ± 2.2	3.0 ± 0.3	0.80 ± 0.02	1.90 ± 0.12

Abbreviations: OA – Ophthalmic artery, CRA – central retinal artery, SPCA – short posterior ciliary artery, LPCA – long posterior ciliary artery, PSV – peak systolic velocity, EDV – enddiastolic velocity, RI – resistive index, PI – pulsatility index.

**Table 2:** Influence of latanoprost on retrobulbar hemodynamics. **Influence of latanoprost on ocular perfusion**. Overview of the results from CDI measurements before and one month after regular application of latanoprost: Increase of blood flow velocities were statistically not significant (n = 10). **Bold **printed measures were subjected to statistical testing; all other parameters were observational only.

		PSV (cms^-1^)	EDV (cms^-1^)	RI	PI
OA	before latanoprost	31.7 ± 3.1	4.6 ± 0.9	0.87 ± 0.02	2.59 ± 0.15
	after latanoprost	39.0 ± 10.1	6.2 ± 1.2	0.83 ± 0.01	2.14 ± 0.09
					
CRA	before latanoprost	7.6 ± 0.6	1.5 ± 0.1	0.78 ± 0.01	1.96 ± 0.10
	after latanoprost	8.4 ± 0.9	1.7 ± 0.1	0.79 ± 0.02	1.95 ± 0.13
					
SPCA	before latanoprost	**8.6 ± 1.8**	**1.8 ± 0.2**	0.74 ± 0.03	1.75 ± 0.13
	after latanoprost	**9.5 ± 1.1**	**2.5 ± 0.4**	0.73 ± 0.03	1.60 ± 0.11
					
LPCA	before latanoprost	13.1 ± 1.5	2.5 ± 0.3	0.81 ± 0.02	1.96 ± 0.14
	after latanoprost	13.0 ± 1.9	2.5 ± 0.4	0.78 ± 0.04	1.90 ± 0.21

Abbreviations: OA – Ophthalmic artery, CRA – central retinal artery, SPCA – short posterior ciliary artery, LPCA – long posterior ciliary artery, PSV – peak systolic velocity, EDV – enddiastolic velocity, RI – resistive index, PI – pulsatility index.

**Table 3:** Influence of dorzolamide on retrobulbar hemodynamics. **Ocular perfusion in the dorzolamide group**. CDI measurements obtained from the patient group treated with dorzolamide (n = 12). The time period between between first and second measurement was 3–5 weeks. Data are given as mean ± standard deviation. **Bold **printed measures were subjected to statistical testing; all other parameters were observational only.

		PSV (cms^-1^)	EDV (cms^-1^)	RI	PI
OA	before dorzolamide	29.6 ± 1.2	3.3 ± 0.4	0.89 ± 0.02	2.82 ± 0.25
	after dorzolamide	37.2 ± 3.5	4.4 ± 0.7	0.88 ± 0.02	2.64 ± 0.20
					
CRA	before dorzolamide	7.3 ± 0.8	1.4 ± 0.1	0.80 ± 0.02	1.98 ± 0.18
	after dorzolamide	8.5 ± 0.8	1.8 ± 0.2	0.78 ± 0.02	1.97 ± 0.24
					
SPCA	before dorzolamide	**8.5 ± 0.8**	**1.9 ± 0.2**	0.77 ± 0.02	1.79 ± 0.11
	after dorzolamide	12.8 ± 1.4 *	**2.2 ± 0.2**	0.80 ± 0.03	1.91 ± 0.13
					
LPCA	before dorzolamide	10.9 ± 0.8	2.0 ± 0.3	0.73 ± 0.07	1.80 ± 0.22
	after dorzolamide	16.9 ± 1.7	2.9 ± 0.3	0.83 ± 0.01	1.95 ± 0.12

Asterisk (*) denotes statistic significance (P < 0.006). Abbreviations: OA – Ophthalmic artery, CRA – central retinal artery, SPCA – short posterior ciliary artery, LPCA – long posterior ciliary artery, PSV – peak systolic velocity, EDV – enddiastolic velocity, RI – resistive index, PI – pulsatility index.

**Table 4:** CDI measurements in the control group. **Ocular perfusion in the control group**. CDI measurements obtained from the control group (n = 8). The time period between between first and second measurement was 3–5 weeks. Local and systemic medication was not changed between the two examinations of the patient. Data are given as mean ± standard deviation. **Bold **printed measures were subjected to statistical testing; all other parameters were observational only.

		PSV (cms^-1^)	EDV (cms^-1^)	RI	PI
OA	first examination	29.3 ± 2.6	3.7 ± 0.6	0.87 ± 0.02	2.57 ± 0.29
	second examination	30.6 ± 3.2	5.4 ± 1.0	0.83 ± 0.02	2.08 ± 0.20
					
CRA	first examination	8.2 ± 1.5	1.8 ± 0.4	0.76 ± 0.03	1.93 ± 0.19
	second examination	7.5 ± 0.9	1.4 ± 0.1	0.80 ± 0.03	2.14 ± 0.21
					
SPCA	first examination	**10.8 ± 1.6**	**2.6 ± 0.5**	0.75 ± 0.02	1.76 ± 0.17
	second examination	**10.1 ± 2.4**	**2.5 ± 0.6**	0.75 ± 0.02	1.55 ± 0.09
					
LPCA	first examination	18.0 ± 3.8	3.1 ± 0.8	0.82 ± 0.04	2.01 ± 0.21
	second examination	18.0 ± 4.0	3.6 ± 0.9	0.77 ± 0.01	1.69 ± 0.12

Abbreviations: OA – Ophthalmic artery, CRA – central retinal artery, SPCA – short posterior ciliary artery, LPCA – long posterior ciliary artery, PSV – peak systolic velocity, EDV – enddiastolic velocity, RI – resistive index, PI – pulsatility index.
